# Metadta: a Stata command for meta-analysis and meta-regression of diagnostic test accuracy data – a tutorial

**DOI:** 10.1186/s13690-021-00747-5

**Published:** 2022-03-29

**Authors:** Victoria Nyawira Nyaga, Marc Arbyn

**Affiliations:** Unit of Cancer Epidemiology - Belgian Cancer Centre, Sciensano, Juliette Wytsmanstraat 14, 1050 Brussels, Belgium

**Keywords:** Meta-analysis, Meta-regression, Diagnostic test accuracy, Stata, *Metadta*

## Abstract

**Background:**

Although statistical procedures for pooling of several epidemiological metrics are generally available in statistical packages, those for meta-analysis of diagnostic test accuracy studies including options for multivariate regression are lacking. Fitting regression models and the processing of the estimates often entails lengthy and tedious calculations. Therefore, packaging appropriate statistical procedures in a robust and user-friendly program is of great interest to the scientific community.

**Methods:**

*metadta* is a statistical program for pooling of diagnostic accuracy test data in Stata. It implements both the bivariate random-effects and the fixed-effects model, allows for meta-regression, and presents the results in tables, a forest plot and/or summary receiver operating characteristic (SROC) plot. For a model without covariates, it quantifies the unexplained heterogeneity due to between-study variation using an I^2^ statistic that accounts for the mean-variance relationship and the correlation between sensitivity and specificity. To demonstrate *metadta,* we applied the program on two published meta-analyses on: 1) the sensitivity and specificity of cytology and other markers including telomerase for primary diagnosis of bladder cancer, and 2) the accuracy of human papillomavirus (HPV) testing on self-collected versus clinician-collected samples to detect cervical precancer.

**Results:**

Without requiring a continuity correction, the pooled sensitivity and specificity generated by *metadta* of telomerase for the diagnosis of primary bladder cancer was 0.77 [95% CI, 0.70, 0.82] and 0.91 [95% CI, 0.75, 0.97] respectively. *Metadta* also allowed to assess the relative accuracy of HPV testing on self- versus clinician-taken specimens using data from comparative studies conducted in different clinical settings. The analysis showed that HPV testing with target-amplification assays on self-samples was as sensitive as on clinician-samples in detecting cervical pre-cancer irrespective of the clinical setting.

**Conclusion:**

The *metadta* program implements state of art statistical procedures in an attempt to close the gap between methodological statisticians and systematic reviewers. We expect the program to popularize the use of appropriate statistical methods for diagnostic meta-analysis further.

## Background

Meta-analysis of diagnostic test accuracy (DTA) studies using approximate methods such as the normal-normal model has several challenges. These include poor statistical properties when sensitivity and/or specificity are close to the margins i.e. 0/1, when the sample sizes or when the number of studies are small. Moreover, the sample variance of sensitivity/specificity is a function of the sample mean and ignoring this mean-variance relationship may bias the summary estimate and its variance. Generalized linear mixed models (GLMM) [1] are therefore recommended [2]. These models are relatively complex requiring expertise both in GLMMs and statistical programming. Scientists in the fields of public health, epidemiology or clinical research often do not have advanced statistical and/or programming skills. Hence, availability and dissemination of appropriate and optimal statistical methods in a robust and user-friendly program is quintessential.

The two most commonly used statistical models for pooling of DTA data are the hierarchical summary receiver operating characteristic model (HSROC) [3] and the bivariate random-effects meta-analysis model (BRMA) [2]. The two models incorporate covariates differently though they have been shown to be equivalent when no covariates are included [4].

The proportion of total unexplained variation due to between-study heterogeneity is usually quantified using the I^2^ statistic by Higgins and Thompson [5]. The statistic is based on the normal-normal model and was defined for univariate meta-analysis. Therefore, in meta-analysis of DTA separate statistics for sensitivity and specificity are computed. The fact that diagnostic data sets are binomial implies that the within-study variance in sensitivity and specificity parameters is a function of the mean parameters. Hence, heterogeneity statistics based on the normal-normal model tend to underestimate the expected value of the within-study variance resulting in high values of I^2^. This could lead to an incorrect conclusion of very high heterogeneity [6].

Zhou and Dendukuri [6] proposed a univariate I^2^ statistic that accounts for the mean-variance relationship across studies. They extended the statistic to account for the correlation between sensitivity and specificity yielding a joint measure of heterogeneity. In a simulation study, they showed that their I^2^ statistic almost always resulted in much lower between-study heterogeneity estimates than the I^2^ by Higgins and Thompson [5].

On interpreting the I^2^, higher values indicate higher between-study heterogeneity across the studies compared to the expected within-study variability.

The reasons for the substantial heterogeneity in the null mixed-effects model can be explored by relating study level covariates to the latent sensitivity and specificity. This is called meta-regression.

There are two Stata commands for meta-analysis of DTA. The *metandi* [7] command fits both the HSROC and the BRMA model. Its output includes a table of the summary accuracy measures and a graph with the SROC curve, the summary point and its confidence region and prediction region. The command does not allow meta-regression.

*midas* [8] is another Stata command. It implements the BRMA only. It produces more graphical output; to explore goodness of fit, publication bias and other precision-related biases. The command only allows for univariate meta-regression with only one covariate and uses the I^2^ statistic based on the normal-normal model.

In this paper, we demonstrate a new Stata command *metadta* which implements the bivariate random-effects and the univariate fixed-effects model as a special case of the bivariate model. The command also allows for univariate and bivariate meta-regression. The results are reported in tables, forest plots and/or SROC plots or cross-hairs. A forest plot of relative sensitivity and specificity can be displayed when data are from comparative or paired studies. For the model without covariates, it quantifies the between-study heterogeneity using the I^2^ statistics by Zhou & Dendukuri [6].

## Methods

### Data structure

Data from DTA studies usually result from a 2 × 2 cross-tabulation of index versus reference test results (see Table 1). The data in the four cells represent the true positive (TP), false positive (FP), true negative (TN), and false negative (FN). The sum of TP and FN is the total with disease, and the sum of TN and FP the total without disease.

**Table 1:** Cross-tabulation of index test results by the disease status in study *i*

		Disease status	
		+	-
Index test	+	True positive (TP) = Y_i1_	False Positive (FP)
	-	False Negative (FN)	True Negative (TN) = Y_i2_
	Total	Diseased = N_i1_	Non-diseased = N_i2_

The command provides statistical procedures for data sets from independent, comparative and paired DTA studies.

Independent studies contribute only one 2 × 2 cross-table and each row in the data set has data from a different study.

From a comparative study, there will be two 2 × 2 cross-tables, one for the index test and the second for the comparator test. Each study contributes two rows to the data set, one for the index test and another for the comparator test. The index and the comparator test should be the same in all the studies.

Paired studies have at least a pair of the 2 × 2 cross-tables. The data for the index and comparator test is on the same row and a study can contribute more than one row to the data set. Unlike data from comparative studies, the index and the comparator tests do not need to be the same. However, it is imperative that the comparator tests are similar in order to obtain correct model estimates. The data set should include at least two index tests.

### The logistic regression model

Consider a meta-analysis of K studies. For a study *i* (i = 1, …, K), let Y_i1_ be the number of true positive, Y_i2_ be the number of true negatives, N_i1_ the total number of subjects with the disease, and N_i2_ the total number of subjects without the disease.

Suppose there are Q study level covariates, the fixed-effects model is formulated as follows;

*Y*_*ij*_~*binomial* (*p*_*ij*_, *N*_*ij*_) for *i* = 1, …, *K* and *j* = 1, 2,

$$ {p}_{ij}=\frac{\exp \left({\beta}_0^j+{\beta}_1^j{X}_{ij}^1\dots {\beta}_P^j{X}_{ij}^Q\right)}{1+\exp \left({\beta}_0^j+{\beta}_1^j{X}_{ij}^1\dots {\beta}_P^j{X}_{ij}^Q\right)}, $$

where *p*_*i*1_ and *p*_*i*2_ are parameters denoting the unobserved sensitivity and specificity in study *i* respectively. ${\beta}_0^j$ are log-odds while ${\beta}_1^j$ … ${\beta}_Q^j$ are log odds ratios. ${X}_{ij}^q$ is the value of the *q’th* covariate in study *i* for logit sensitivity(j = 1) and logit specificity (j = 2).

The random-effects model has (un) correlated random components in the mean predictor. It is expressed as follows;

$$ {p}_{ij}=\pi (x)=\frac{\exp \left({\beta}_0^j+{\beta}_1^j{X}_{ij}^1\dots {\beta}_P^j{X}_{ij}^P+{\delta}_{ij}\right)}{1+\exp \left({\beta}_0^j+{\beta}_1^j{X}_{ij}^1\dots {\beta}_P^j{X}_{ij}^P+{\delta}_{ij}\right)}, $$

$$ \left(\genfrac{}{}{0pt}{}{\delta_{i1}}{\delta_{i2}}\right)\sim N\left(\left(\genfrac{}{}{0pt}{}{0}{0}\right)\right.,\left.\boldsymbol{\varSigma} \right), $$

where *δ*_*ij*_ are the study-specific random-effects for the logit sensitivity(j = 1) and the logit specificity (j = 2). The variation in the two random effects and their correlation is represented by ***Σ*.** The structure of ***Σ*** can be any of the four variance-covariance matrices: unstructured $$ \left(\begin{array}{cc}{\tau}_1^2& {\tau}_{12}\\ {}{\tau}_{12}& {\tau}_2^2\end{array}\right) $$, independent $$ \left(\begin{array}{cc}{\tau}_1^2& 0\\ {}0& {\tau}_2^2\end{array}\right) $$, exchangeable $$ \left(\begin{array}{cc}{\tau}^2& {\tau}_{12}\\ {}{\tau}_{12}& {\tau}^2\end{array}\right) $$ or identity $$ \left(\begin{array}{cc}{\tau}^2& 0\\ {}0& {\tau}^2\end{array}\right) $$. The BRMA imposes the unstructured variance-covariance matrix. It makes the most relaxed assumption about the co-variation of the random effects but has the most number of parameters, i.e. 3 distinct parameters. Adding ${\beta}_0^1$ and ${\beta}_0^2$, it implies that there needs five parameters to be identified in the null model. Hence, at least five studies would be required to enable parameter identification. Other structures are more restrictive but require less studies (at least 3) for identifiability.

When a random effects model is fitted to the data set, a log-likelihood ratio (LR) test is conducted to compare it with the fixed effects model. The reported *p*-value is an upper bound of the actual p-value because this hypothesis test is on the boundary of the parameter space of the variance parameters.

The models presented above are applied when the data are from independent studies. With data from comparative or paired studies, the linear predictor is modified to account for the dependence introduced by the “repeated measurements” per study. This modification is critical in the interpretation of the random variation in the data as well as in obtaining valid model-based inference for the mean structure.

When there are more than one covariates, the fixed effects component in the linear predictor can be extended to include interaction terms between the first covariate and the remaining covariates. A LR test can then be conducted comparing the model with and without the interaction terms. This would give an answer as to whether the interaction terms are necessary in the model.

### Summary tables

We report the marginal summary estimates and not the direct model parameter estimates. The marginal sensitivity and specificity are averages of the predicted probabilities from the model. They are said to be standardized to the distribution of the covariates [7, 9]. The model-adjusted probability ratios are computed as a ratio of the marginal probabilities.

### Forest plot

The command presents five different confidence intervals (CI) for the study-specific sensitivity and specificity; the Wald, Wilson, Agresti-Coull, Jeffreys, and exact confidence intervals. The exact confidence intervals are displayed by default.

With data set from comparative and paired studies, the Koopman score confidence intervals [10] for the study-specific relative sensitivity and specificity are calculated.

### SROC plot

Separate summary points and their confidence regions and/or prediction regions are presented when there is only one categorical covariate in the model. When the number of studies is insufficient to fit the random-effects model or when the fixed-effects model is explicitly applied, cross-hairs indicating the confidence intervals of the summary estimates are presented instead. In presence of more than one covariate, the SROC plot presents only the overall summary point and the corresponding confidence region and/or prediction region. When focus of is on the relative diagnostic accuracy i.e. when the forest plot presents the relative sensitivity and specificity, the SROC plot is not presented.

When plotting the SROC curve, the program restricts the curve to the range of the specificities in the dataset.

### Software installation

The *metadta* command was developed in Stata 14.2. The program along with the help files and three demonstration datasets are publicly available for downloading at https://ideas.repec.org/c/boc/bocode/s458794.html. When connected to the internet, the command can be directly installed within Stata by typing **ssc install metadta.**

### Syntax


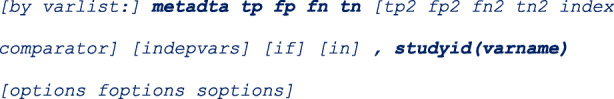


The *metadta* command requires five main arguments to run. These are; ***tp fp fn tn*** indicating the four outcome variables from the 2 × 2 cross-tabulation in Table 1. The fifth argument **studyid(varname)** is the study identifier. The other arguments in italics are optional.

Categorical variables in the data set should be string variables otherwise, the command will treat them as continuous variables. The Stata command *decode* can be used to make a factor variable into a string variable. The covariates names should not contain the underscore(_) character. If present, the program terminates because the underscore character is reserved in the program. If some of the covariates names contain the underscore character, the Stata command *rename* can be used to give those covariates different names. The options *[options foptions soptions]* could be;


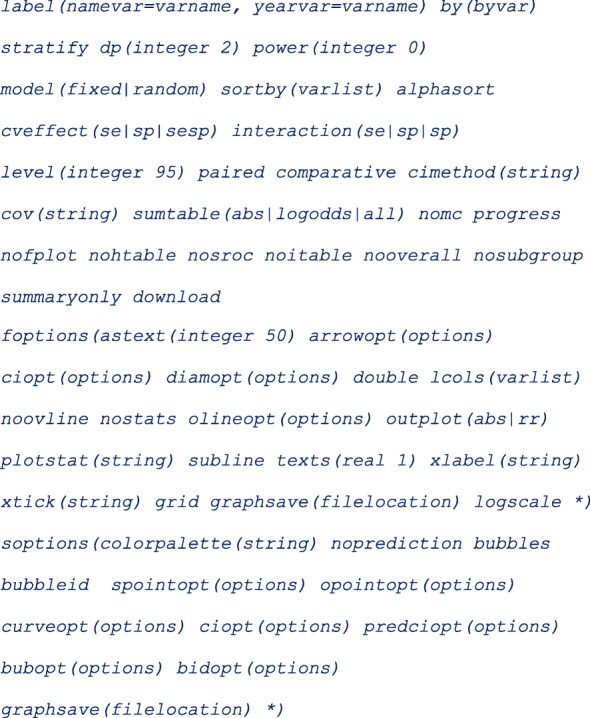


Once installed, typing **help metadta** should display the help window. The help file provides a detailed description of all the command options. Some of the options worth mentioning here include;

*by varlist:* allows separate but similar meta-analyses for each level of the *by* variable or each combination of the *by varlist* variables. The results are presented in separate summary tables, forest plots and SROC plots. If it is desired to perform separate analysis but present the results in one forest plot and/or one SROC plot, one should specify the options *stratify* and *by(byvar)* simultaneously. The two options *by varlist:* and *by(byvar)* should not be confused for each other.

*indepvars* indicates one or more variables to be used as covariates. They should be string/characters for categorical variables and/or numeric for continuous variables. The variable names should not contain underscores, it is reserved in the program.

*comparative* indicates whether the data supplied are from comparative studies. This option requires the first covariate specified to be categorical with two levels, one for the index and the comparator test or level.

*paired* indicates whether the data supplied are from paired studies. This option requires at least 11 variables (including the study identifier) in the data set in the following order *tp1 fp1 fn1 tn1 tp2 fp2 fn2 tn2 index comparator*.

* are options native in Stata to change the graphics aesthetics. The default plots are already visually appealing but can be optimized by *soptions()* and *foptions()* for the SROC plot and the forest plot, respectively.

### Application

#### Example one – random-effects model with no covariates

Glas et al. [11] conducted a meta-analysis to assess the sensitivity and specificity of urine based markers such as telomerase for diagnosis of primary bladder cancer. This data set of 10 studies is provided along with the installation files.


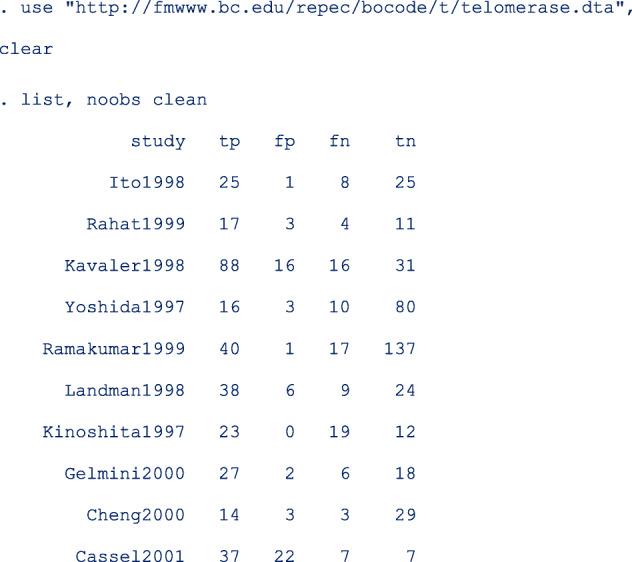


Because the seventh study **Kinoshita1997** had an estimated specificity equal to one (**fp = 0**), the authors needed to use a continuity correction of 0.5 to enable parameter estimation with the bivariate normal-normal model. They reported that telomerase had a sensitivity and specificity of 0.75 [95% CI, 0.66, 0.74] and 0.86 [95% CI, 0.71, 0.94] respectively. They concluded that telomerase was not sensitive enough to be recommended for daily use.


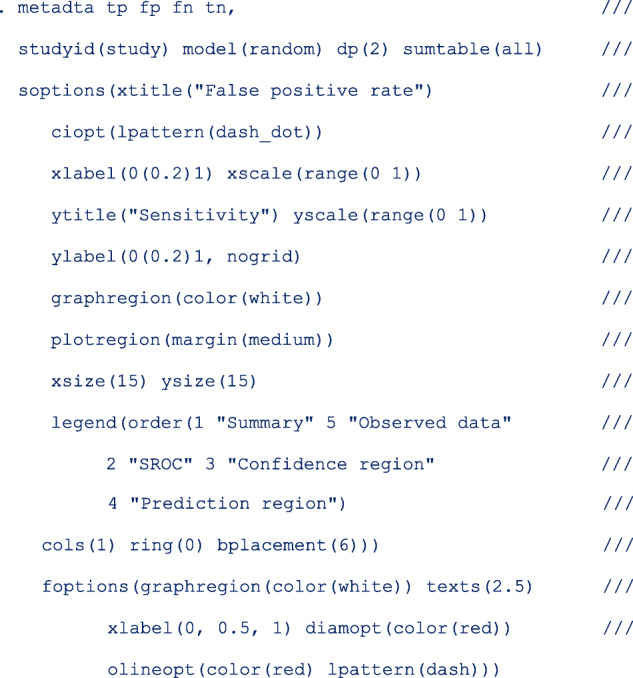


Apart from the required main arguments, we also specified the option model (random) to request for the random-effects model. This option is redundant since the command fits the random-effects by default. When the program detects that the number of studies is less than 3, the fixed-effects model is fitted instead. *dp(2)* requests the results of all estimates to be displayed with 2 decimal places (except the *p*-values for which the decimals places are fixed at 4). The options in *soptions()* and *foptions()* refined the appearance of the forest and SROC plots.

The first part of the output displays the symbolic representation of the fitted model, the number of observations and the number of studies in the meta-analysis as shown below;


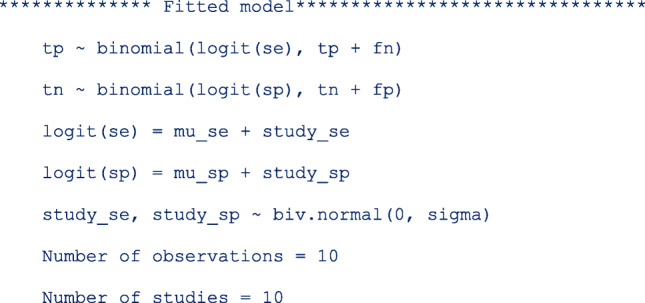


The next part of the output presents the heterogeneity statistics. This table can be suppressed by the option nohtable. By default, the unstructured covariance matrix is imposed. The correlation (rho) between sensitivity and specificity on the logit scale is − 1. There is more heterogeneity in specificity (*σ*^2^ = 3.32, *I*^2^ = 60.29%) than in sensitivity *σ*^2^ = 0.18, *I*^2^ = 50.62 % ).

Despite presence of heterogeneity in both dimensions, it may be surprising that the bivariate I^2 = 0.02. This is because the generalized between-study variance goes to zero with (nearly) perfect correlation (*rho* = − 1.00), and the lower the bivariate I^2. The generalized between-study variance was < 0.0001. It summarizes the variance in both logit sensitivity and specificity while accounting for the correlation between them.

The *p*-value of the LR test comparing the fitted random-effects to a fixed-effects model is < 0.0001. This indicates that the random-effects is a better fit to the data. The test has three degrees of freedom since the unstructured covariance matrix has 3 parameters.


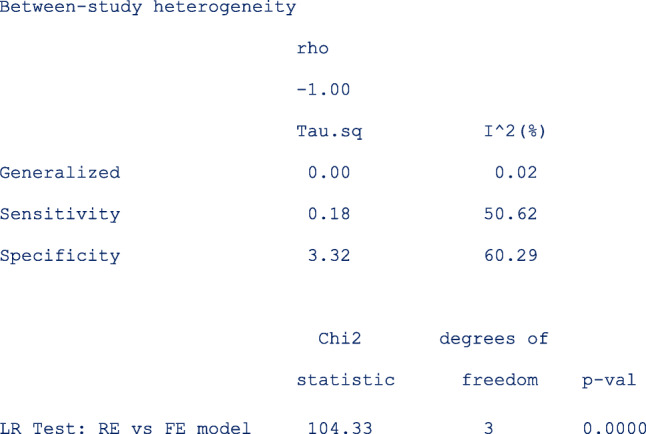


*sumtable (all)* requested for all available summary tables, i.e. summary estimates on the log odds and the probability scale. These are presented as follows;


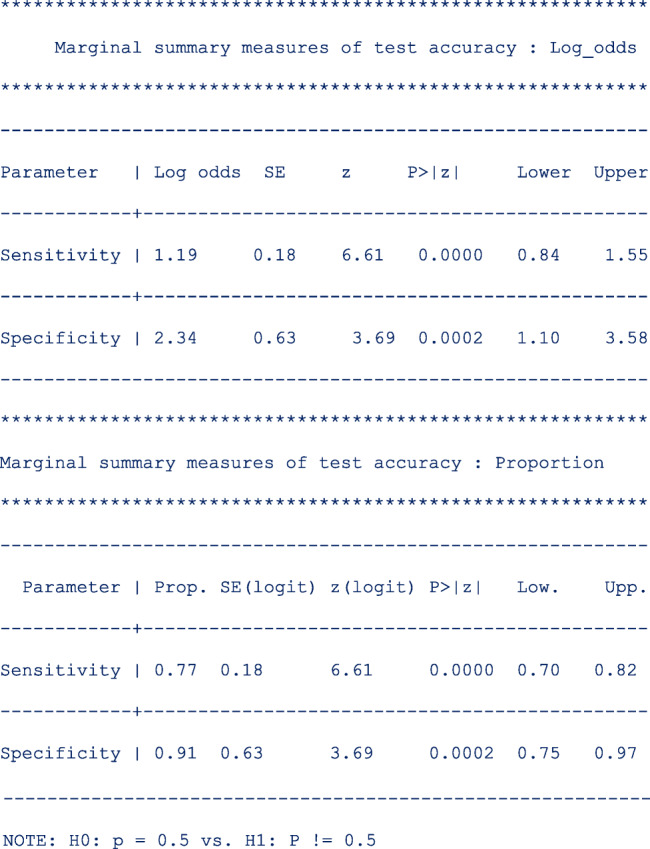


The mean logit sensitivity and specificity are 1.19 [95% CI, 084, 1.55] and 2.34 [95% CI, 1.10, 3.58]. The *p*-values from testing whether the logit sensitivity or logit specificity is 0 are both < 0.01. Thus the logits are significantly different from zero.

The second table presents the same summary statistics but on the probability scale. The standard errors, the z-statistic and the p-values are reported on the logit scale. Translated in the probability scale, the *p*-values are from testing whether the mean sensitivity/specificity is 0.5. If needed, one can use the delta method to compute the standard errors on the probability scale.

The pooled sensitivity and specificity of telomerase in urine as a tumour marker for the diagnosis of primary bladder cancer was 0.77 [95% CI, 0.70, 0.82] and 0.91 [95% CI, 0.75, 0.97] respectively. Our results are different from the original publication because we use the logistic-normal model while they used the normal-normal model.

The third table below presents the study-specific and summary sensitivity and specificity and their corresponding 95% exact CI.


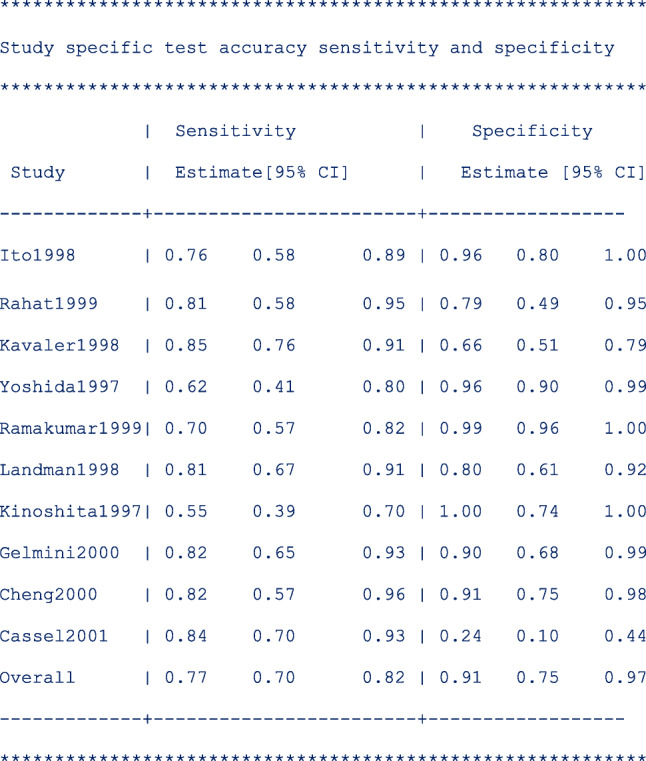


**Fig. 1:**
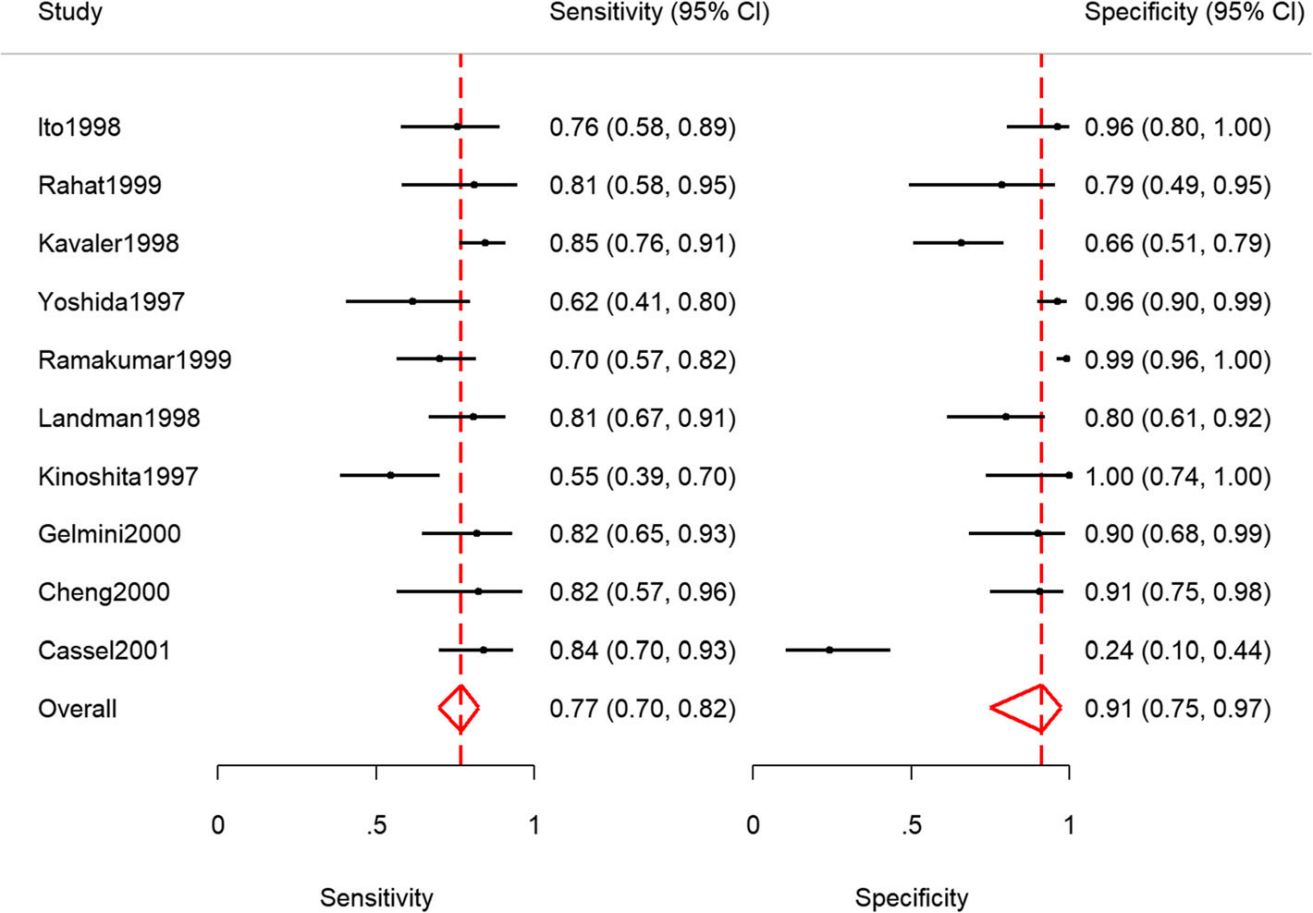
Forest plot - meta-analysis of diagnostic accuracy of telomerase for the diagnosis of bladder cancer

**Fig. 2:**
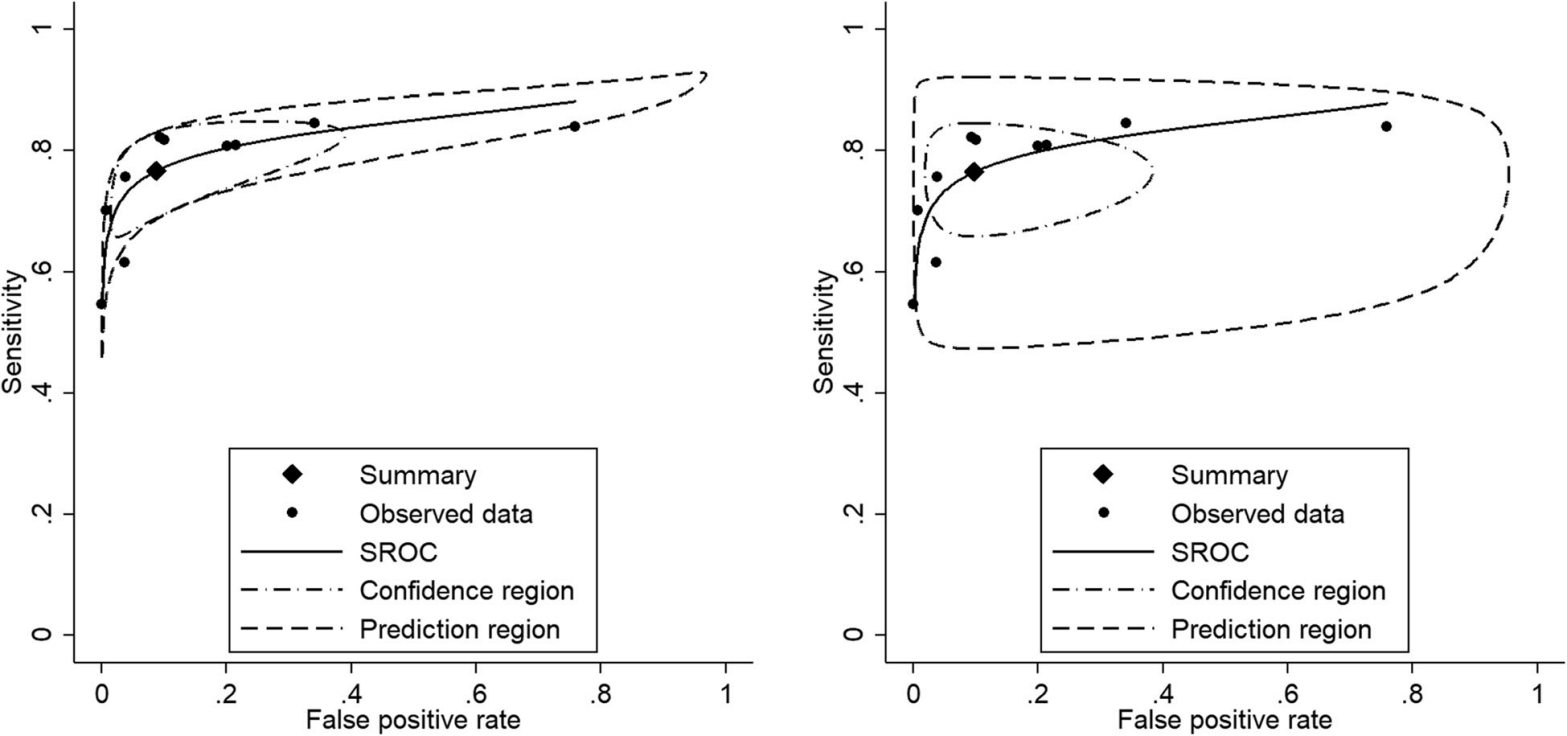
SROC plots - meta-analysis of diagnostic accuracy of telomerase for the diagnosis of bladder cancer. Left: the unstructured covariance and right: the independence covariance

Figures 1 and 2 (left) presents the forest and the SROC plots respectively. The program preserves the order in the data set. Say it is preferred to order the studies by year of publication, a variable with the year of publication (say **year**) should be included in the data set. The option  *sortby(year)* then instructs the program to re-order the data set.

#### Model comparison - covariance structures

To impose a different covariance structure, say independence and select the most parsimonious model we proceeds as follows;

1. First restore the model estimates by typing **estimates restore metadta_modest** (the estimates of the current model are always stored as **metadta_modest**). Once restored, use the command **estat ic** to display the Akaike information criteria (AIC) and Bayesian information criteria (BIC) [12]. The output is;

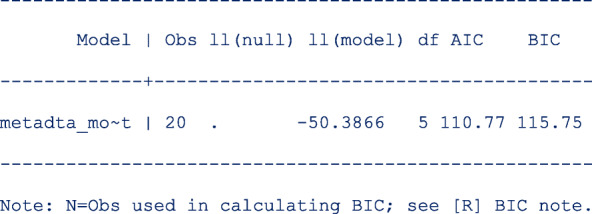
2. Use the command **estimates store** to store the estimates for later use under a different name, say unstructured. i.e. **estimates store unstructured**.
3. Fit a new model imposing independence between logit sensitivity and logit specificity with the option  *cov(independent)*.
4. Repeat step 1 above to be able to display the information criteria. The output is;

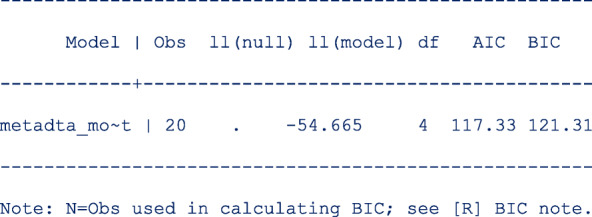

The models compared using the information criteria do not need to be nested but should use the same data. The model with a smaller information criterion fits the data better. From the output in steps 1 and 4, the model with the unstructured covariance matrix fits the data better since both the AIC and the BIC are lower.

Sometimes the AIC and BIC can give conflicting conclusions. In this example, both give the same conclusion. The difference between AIC and BIC is in measuring the model complexity. Model complexity is measured either as 2*q or ln (K)*q, where q is the number of parameters estimated in the model and K is the number of observations in the data set. Explicitly,

AIC = − 2 x ln (likelihood) + 2 x q.

BIC = − 2 x ln (likelihood) + ln(K) x q.

By overweighting the model complexity, the BIC is more conservative than AIC.

#### Implication of assuming the independence covariance structure

The pooled sensitivity and specificity under the independence assumption is 0.77 [95% CI, 0.70, 0.82] and 0.90 [95% CI, 0.75, 0.97]. The pooled estimates are very similar to those from the first model.

However, the heterogeneity statistics are much more different; logit sensitivity (*σ*^2^ = 0.15, *I*^2^ = 46.81%) and logit specificity (*σ*^2^ = 2.75, *I*^2^ = 65.01%). The estimate for the generalized variability is much higher (*σ*^2^ = 0.43, *I*^2^ = 56.11%) because there is (assumed) no correlation.

When the assumed covariance structure is far from ‘correct’, the estimates for the mean always tend to be consistent. However, the confidence intervals, confidence region and prediction region might be wider. In Fig. 2 (right), assuming no correlation between the logit sensitivity and the logit specificity yields wider regions.

### Example two – random-effects meta-regression

Arbyn et al. [13] published a meta-analysis on the accuracy of HPV testing on self-collected versus clinician-collected samples. In the review, they sought to find whether a HPV test on a vaginal self-sample was as accurate as on a cervical sample taken by a clinician to detect cervical precancer (cervical intraepithelial neoplasia of grade 2 or worse [CIN2+]). *metandi* [7] was used to generate the pooled absolute sensitivity and specificity and *metadas* [14] was used to obtain the relative sensitivity and specificity (self-sample vs clinician sample) by the test amplification method (signal or target amplification).

The studies included in the meta-analysis had been conducted in three clinical settings: 1) cervical cancer screening, 2) testing of high-risk women, and 3) colposcopy, where women were referred to because of previous positive screening results.

Other information on the study participants, name of the test used, the sampling device were recorded also.

We use a sample of the published data set where studies applied the same test on a self-sample and a clinician-sample from the same women (comparative studies). The first 10 of the 60 observations are as below;


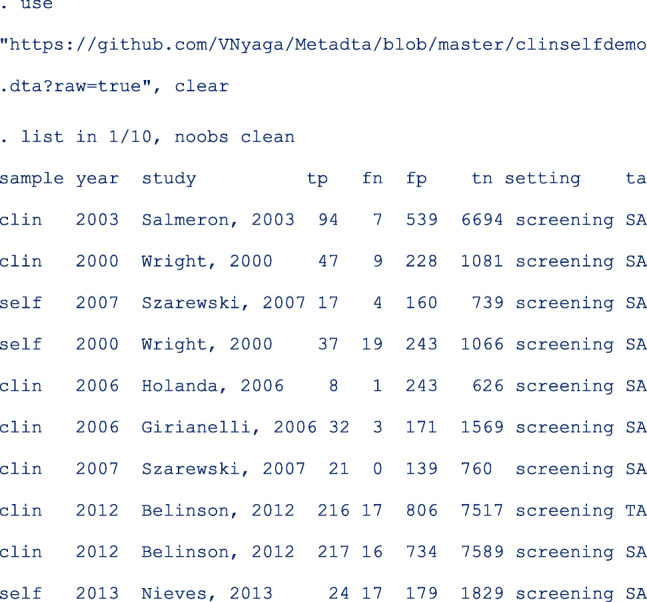


where **sample**, **setting** and **ta** are categorical (characters/string) covariates each with two values. **sample** is **clin** or **self** in the clinician-sample and self-sample respectively. **Year** indicates the year of study publication. **setting** identifies the clinical setting of the study with values screening or follow-up. **ta** is **TA** for a target amplified test or **SA** for a signal amplified test.

#### Exploratory analysis

We investigate whether the absolute sensitivity and specificity of the self- and clinician-collected sample differ by the test amplification method and the setting of the study. To do this, we fit four models for each combination of the categories in setting and ta with sample as a covariate.

The command preserves the order of the data and therefore, the values in each categorical variable are decoded based on the first-come-first-assignment. The Stata command **gsort** sorts the data alphabetically such that the base level for sample is clin and the second level is self.


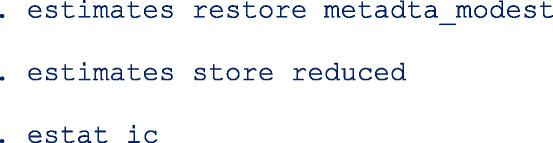


The values in the categorical variables can decoded based on the alphabetic order (A to Z) while still preserving the order in the data set with the option alphasort. The first value of the categorical covariates used in the model are assigned the base levels.

The code to fit the first model is as follows;


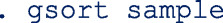


The option *noitable* suppresses the table with the study-specific estimates, and *sumtable (abs rr)* requests to display the absolute and relative specificity and relative sensitivity only (the summary table of the log odds will be suppressed). In this analysis, we are not interested in the SROC plot and suppress its display with the option *nosroc*.

From the output below, there are 6 observations from 3 studies in the meta-analysis.


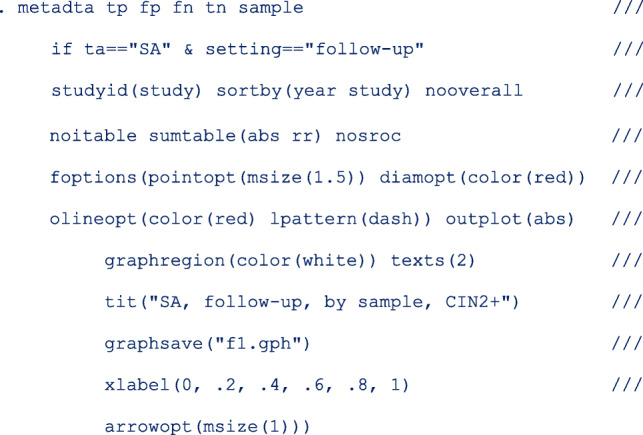


In this model, the base level in **sample** is **clin**.


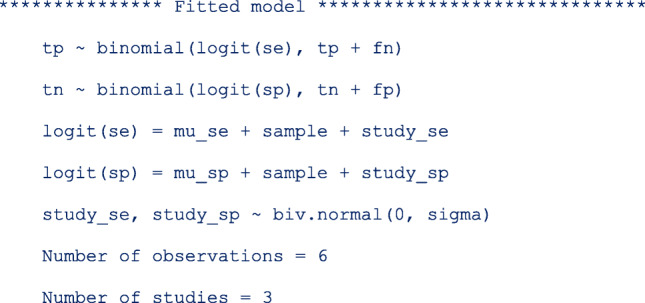


The next part of the output below displays the linear predictor representation of two simpler models fitted to the data set for model comparison. The fitting of the additional models could take some time especially in more complex or larger models. If not necessary, they can be skipped with the option *nomc*. The two simpler models leave out the **sample** term in each of the two predictor equations.


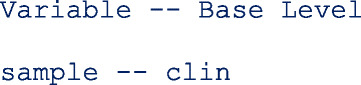


In meta-regression, the I^2^ statistics are not calculated. From the output below, there is more heterogeneity on the logit sensitivity (Tau.sq. = 0.56) than on the logit specificity (Tau.sq. = 0.06). The generalized I^2^ is even less (Tau.sq. = 0.03) after accounting for the correlation (rho = 0.13) between the logits. Compared to the model with fixed study effects, the model with random study effects fits the data better (*p* = 0.0018).


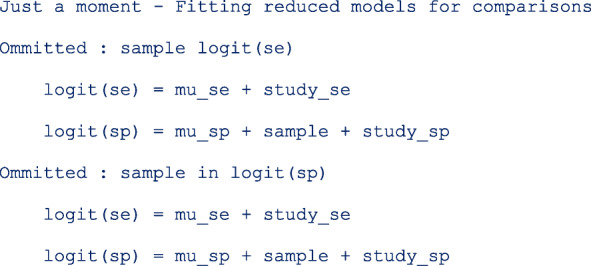


The table below shows the pooled sensitivity and specificity of signal amplified HPV tests on self- and clinician-samples in the follow-up setting:


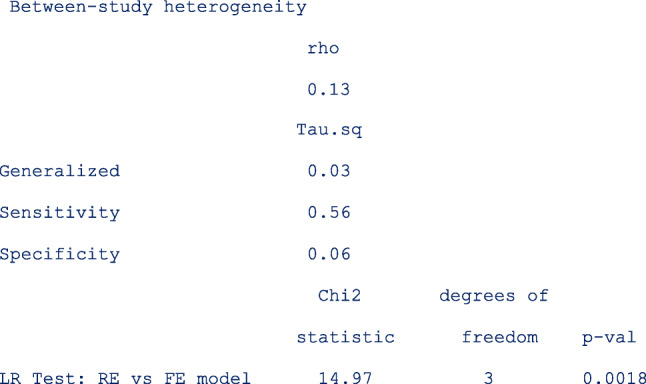


The next table (below) shows the pooled relative sensitivity and specificity of signal amplified HPV tests on self- vs clinician-samples in follow-up setting;


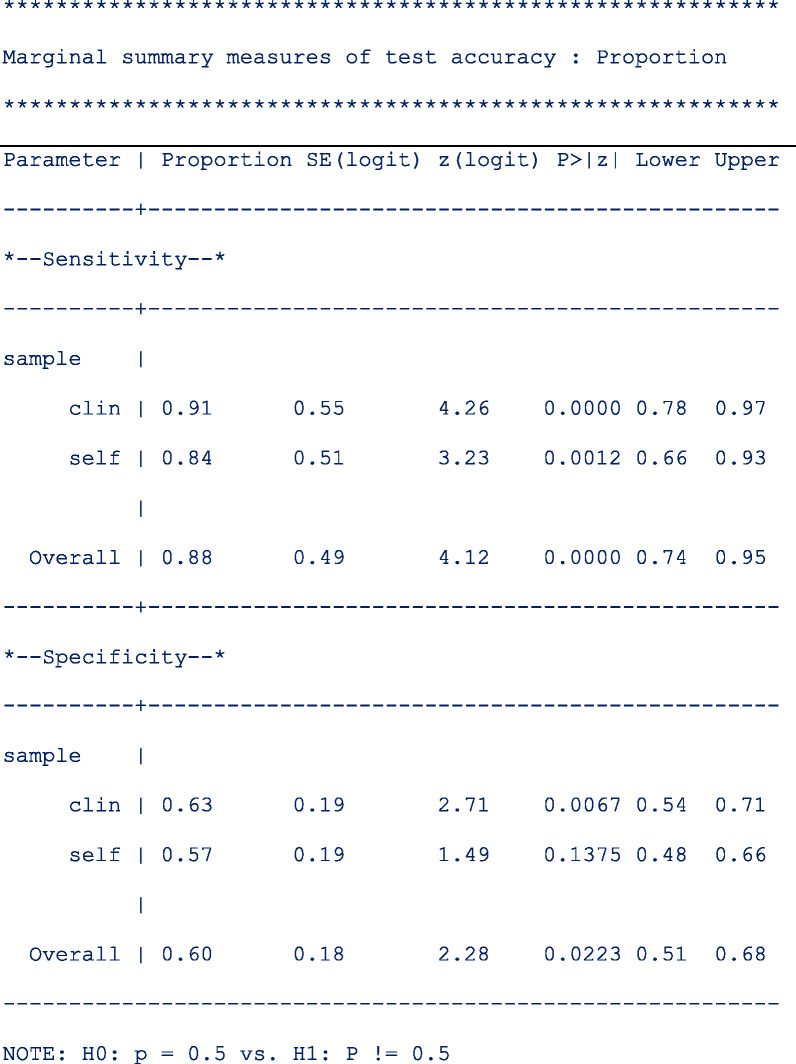


From the output above, the signal amplified tests on self and clinician samples have similar sensitivity (Rel Ratio = 0.92 [95% CI: 0.81, 1.04]) and specificity (Rel Ratio = 0.91 [95% CI, 0.81, 1.02]) in the follow-up setting.

The output below shows the model comparisons results. The results indicate that the model without sample on the linear predictor for logit sensitivity or logit specificity is more parsimonious (*p*-values are > 0.05 in both cases). The conclusion here from the LR test is essentially the same as from the table with the relative diagnostic accuracy estimates.


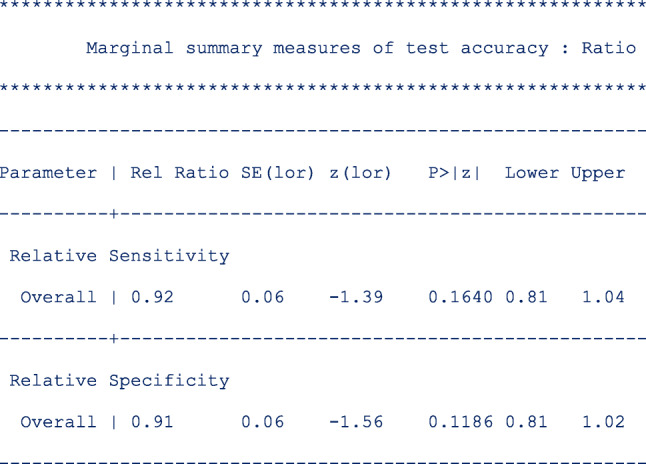


**Fig. 3:**
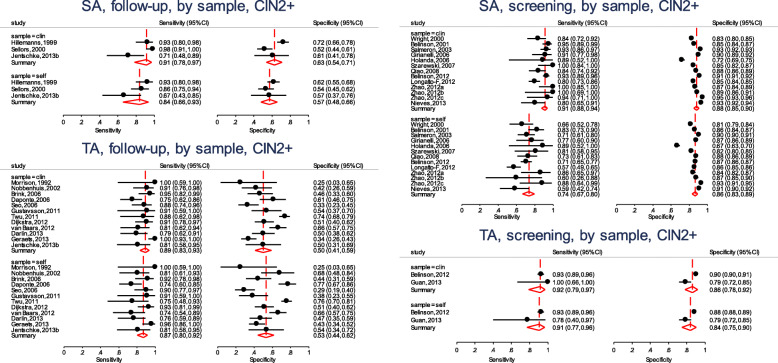
Forest plots - absolute accuracy for CIN2+ of HPV testing on self-samples and clinician-samples using signal amplification-based (SA) assays (top) or target amplification-based (TA) assays (bottom) in the follow-up setting (left) and the screening setting (right)

We fit the other three models by changing **if ta==“SA” & setting==“follow-up”** to include the other combinations of the test amplification method and the setting. Figure 3 presents the forest plots from the four fitted models. From the forest plots, the pooled specificity is consistently lower in follow-up settings (range 50–63%) and substantially higher values in the screening setting (range 84–88%) suggesting that the absolute accuracy differed by settings. Our interest however, is in answering whether a HPV test on a vaginal self-sample is as good as on the cervical sample taken by a clinician.

#### Confounding effects

To formally assess the differences by **setting**, we enter a second covariate into the model. In another instance, we do the same for the test amplification method ta. In the command, we indicate that the studies in the data set are comparative with the option comparative and request for a forest plot of the relative specificity and relative sensitivity with option *outplot (rr)* in *foptions()*. The SROC plot is automatically not generated when the option outplot (rr) is specified.

**Fig. 4:**
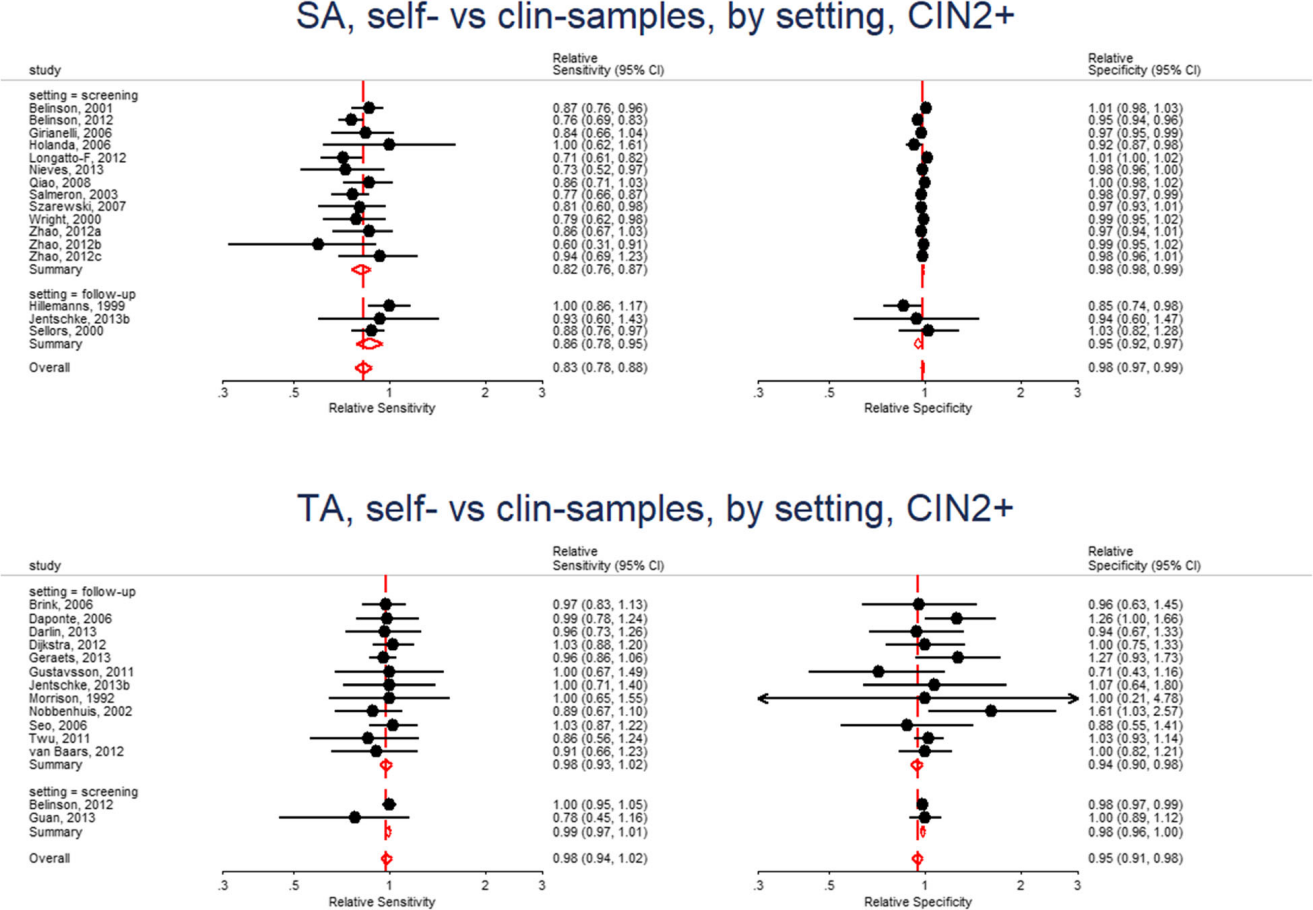
Forest plots - relative accuracy for CIN2+ of HPV testing on self-samples vs clinician-samples using signal amplification-based (SA) assays (top) or target amplification-based (TA) assays (bottom) by setting

In Fig. 4, we observe that the relative sensitivity of signal amplified tests on self- vs clinician- sample is consistently lower than unity irrespective of setting (top left). In contrast, the relative sensitivity of target amplified tests on self- vs clinician-sample consistently includes unity (bottom left) in the screening and follow-up setting. The pooled relative specificity show limited variation by setting.

The findings indicate that it is reasonable to report the “pooled” relative accuracy estimates without regard to setting for a given method of test amplification.

#### Including the interaction terms

The plots in Figs. 3 and 4 suggest that the setting and the test amplification method both significantly influence the absolute accuracy but that the setting has a minor influence on the relative accuracy. To formally examine how setting and ta modify the sensitivity and specificity for CIN2+ of HPV testing on self- and clinician-sample we include the covariates **ta** and **setting** in the model. We also include the interactions terms between sample and ta and between sample and setting by specifying the option *interaction(sesp)*. This option specification instructs the program to add interaction terms on both the linear predictors for logit sensitivity and specificity.


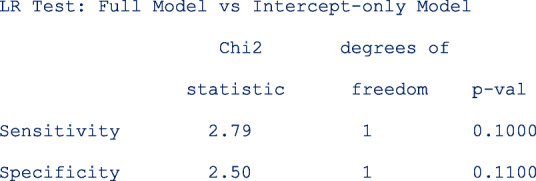


The dataset in the meta-analysis comprised 60 observations from 28 studies.


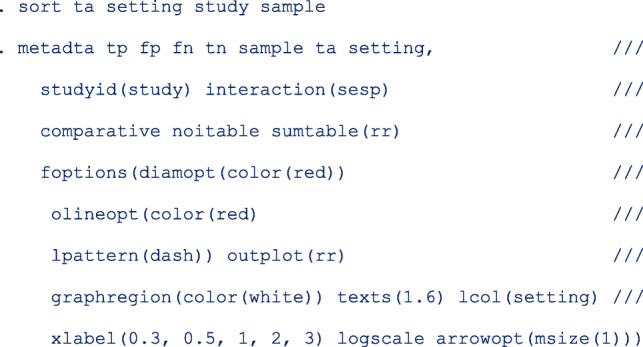


We requested for the diagnostic accuracy estimates with the option *sumtable(rr)* though we do not present the table here. Controlling for the method of test amplification, the relative sensitivity of HPV testing on self- vs. clinician-sample in the screening and follow-up settings are 0.91 [95% CI: 0.86, 0.95] and 0.92 [95% CI: 0.86, 0.98] respectively. The confidence intervals over-lap suggesting that there might be little or no difference in the pooled relative sensitivities. Similarly, controlling for the clinical setting, the relative sensitivity in target-amplified and signal-amplified tests are 0.82 [95% CI: 0.76, 0.89] and 0.98 [95% CI:0.95, 1.01] respectively. The confidence intervals do not overlap suggesting differences by test amplification method. The relative specificity can be interpreted in a similar manner.

Wald-type tests for non-linear hypotheses were conducted to formally test whether the relative sensitivities and relative specificities were similar in all settings and test amplification methods. The results are displayed as follows;


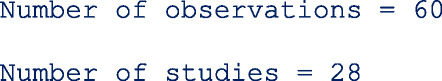


From the output above, each hypothesis test has one degree of freedom because there is only one contrast examined, e.g. for setting, the contrast is **RR.screening = RR.follow-up**.

The results indicate that after controlling for the test amplification method, the relative sensitivities were similar (*p* = 0.7049) in the two clinical settings. In contrast, the pooled relative sensitivities were different (*p* = 0.0001) between the two test amplification methods after controlling for the clinical setting. Furthermore, there were no differences in the pooled relative specificities by clinical setting (*p* = 0.4799) or by test amplification method (*p* = 0.6909) after controlling for the type of test and the clinical setting respectively.

We also tested whether the interaction terms were significant by leaving out one interaction term in each of the predictor equation at a time. The output below indicates that neither of the two interaction terms in the predictor equation for logit specificity are significant.


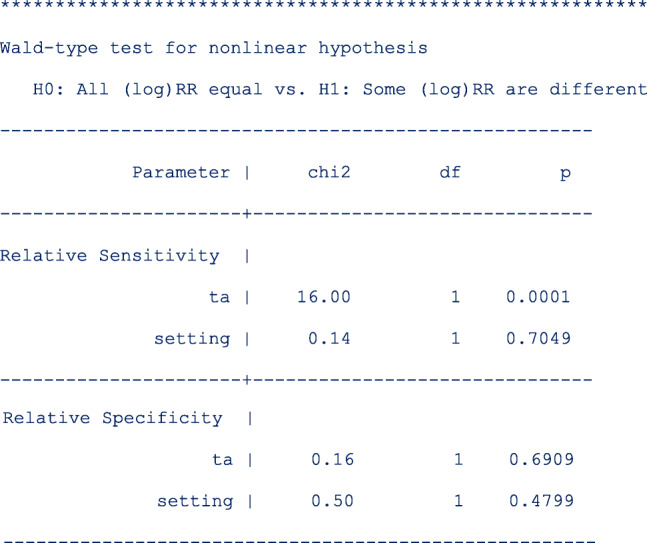


#### Fitting a simpler model

The simpler model without interaction terms in the predictor equation for logit specificity is fitted to the data set with the option *interaction(se)*. The option instructs the program to add the interaction terms only in the predictor equation for logit sensitivity.

Before running the command, we restore and store the model estimates under a different name (say **full**) for use later to compare the current model (*interaction(sesp)*) with the next model (*interaction(se)*).


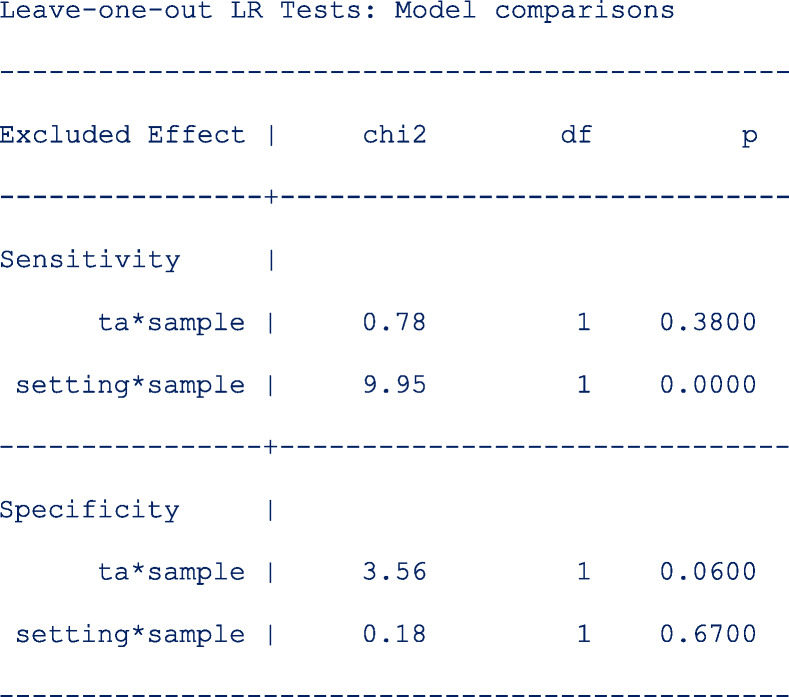


The predictor equations for the model with interaction terms only on the logit sensitivity are as follows;


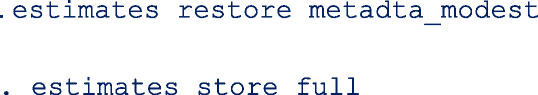


Other simpler models leaving out the interaction terms or the main term from the predictor equation for the logit sensitivity and the logit specificity respectively are also fitted to the data. The model comparison results are as follows;


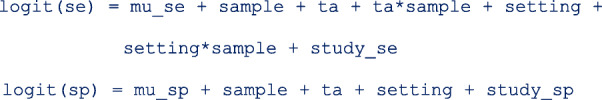


From the output above, leaving out ta*sample (*p* = 0. 3800) or **setting** (p = 0. 3700) from the linear predictor of the logit sensitivity and logit specificity respectively, would yield a more parsimonious model.

We restore and store the estimates under the name **reduced** and request for the AIC and BIC of the current model.


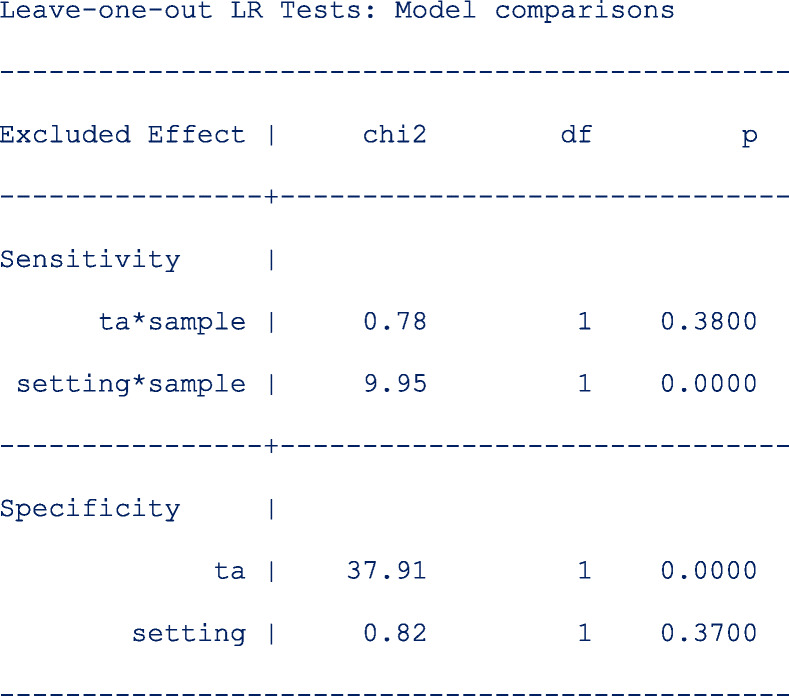


Both the AIC (reduced = 907.6009, full = 908.0111) and BIC (reduced = 943.8383, full = 949.8235) indicate that the reduced model fits the data set slightly better.

As mentioned earlier from **Leave-one-out LR Tests: Model comparisons**, the reduced model could be improved further by removing more terms from the predictor equations. However, the *metadta* program is not flexible to fit a model without ta*sample while keeping setting*sample or a model that includes the interaction terms on the logit sensitivity but leave out the main term for setting in the predictor equation of the logit specificity. Nonetheless, this “fine-tuned” model can be fitted outside *metadta* via the native Stata command *meqrlogit.*

**Fig. 5:**
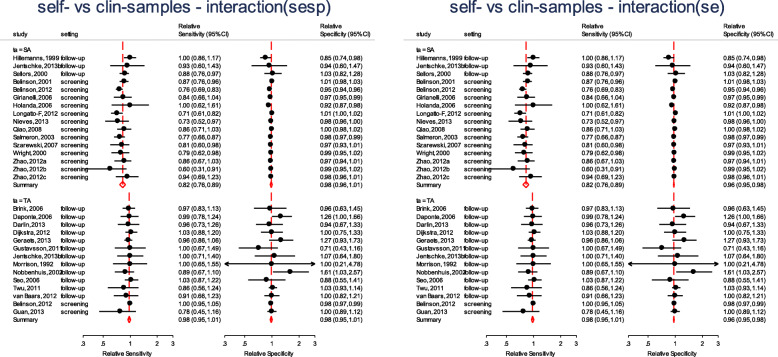
Relative sensitivity and specificity for CIN2+ of HPV testing on self- vs clinician-samples controlling for setting and test amplification method

Figure 5 displays the forest plot from the full (on the left) and the reduced model (on the right). There are differences in the estimates for the pooled relative specificity but not for the pooled relative sensitivity.

The pooled relative diagnostic accuracy estimates from the reduced model in Fig. 5 (right) are very similar to the “stratified” meta-regression results presented in Fig. 4.

## Discussion

This tutorial demonstrated some of the capabilities of *metadta* to perform meta-analysis and meta-regression of DTA studies in Stata. Random-effects models with and without covariates were fitted using logistic regression. The model-adjusted pooled absolute and relative diagnostic accuracy were presented in tables and graphically in forests and/or SROC plots.

We developed m*etadta* to provide advanced statistical procedures for data sets from independent, comparative and paired DTA studies. We encourage users of our program to explore the help file and run the demonstration examples to further familiarize with *metadta*.

With *metadta,* we expect to close the gap between expert methodological statisticians and systematic reviewers and to boost the use of more appropriate methods for meta-analysis and meta-regression of DTA studies even further.

## Data Availability

The *metadta* program was developed in Stata 14.2. The code, the help files used herein are publicly available for download at https://ideas.repec.org/c/boc/bocode/s458794.html.

## Abbreviations

SROC: Summary receiver operating characteristic
HPV: Human papillomavirus
DTA: Diagnostic test accuracy
GLMM: Generalized linear mixed models
HSROC: Hierarchical summary receiver operation characteristic model
BRMA: Bivariate random-effects meta-analysis
TP: True positive
FP: False positive
FN: False negative
TN: True negative
AIC: Akaike information criterion
BIC: Bayesian information criterion
CIN2 +: Cervical intraepithelial neoplasia of grade 2 or worse
TA: Signal-amplification
SA: Test-amplification
