# Correction: Metadta: a Stata command for meta-analysis and meta-regression of diagnostic test accuracy data – a tutorial

**DOI:** 10.1186/s13690-022-00953-9

**Published:** 2022-09-27

**Authors:** Victoria Nyawira Nyaga, Marc Arbyn

**Affiliations:** Unit of Cancer Epidemiology - Belgian Cancer Centre, Sciensano, Juliette Wytsmanstraat 14, 1050 Brussels, Belgium

**Correction: Arch Public Health 80, 95 (2022)**

**https://doi.org/10.1186/s13690-021-00747-5**

Following publication of the original article [1], the author reported that starting from section ‘exploratory analysis’ unfortunately the boxes with the codes have been misplaced, so the wrong code appears in the text.

The original article [1] has been corrected.
